# SELF-REPORTED FRAUD VERSUS OBSERVED VICTIMIZATION INCIDENTS: A RISK FACTOR COMPARISON

**DOI:** 10.1093/geroni/igae098.0721

**Published:** 2024-12-31

**Authors:** Marguerite DeLiema, Siyu Gao, Lynn Langton, M Daniel Brannock

**Affiliations:** University of Minnesota Twin Cities, St. Paul, Minnesota, United States; University of Minnesota, Minneapolis, Minnesota, United States; RTI International, Research Triangle Park, North Carolina, United States; RTI International, Research Triangle Park, North Carolina, United States

## Abstract

According to the age-associated financial vulnerability model, older adults may be more susceptible to scams. Research is needed to identify modifiable risk factors for targeted intervention. We surveyed 934 law enforcement-identified victims of mass marketing scams (mean age=74.7) to determine what characteristics and behaviors are associated with the number of (1) self-reported victimization incidents, and (2) observed victimization incidents. The survey was administered online and by mail. Data on participants’ observed incidents of mail fraud victimization between 2022 and 2023 were provided by the US Postal Inspection Service. Linear regression results indicate that behaviors that increase fraud exposure (e.g., answering calls from unknown numbers, talking with telemarketers, opening all pieces of mail), as well as purchasing lottery tickets, loneliness, and a preference to take financial risks are associated with a greater number of self-reported fraud and exploitation incidents. Identifying as non-Hispanic White and being more active online were protective. Purchasing lottery tickets and greater preference for financial risks were also significantly associated with observed incidents of fraud. Being female and reporting less social engagement were significant for observed incidents only. Based on the difference in significant risk factors between the two models, results indicate that self-reported victimization is an imperfect measure of actual fraud victimization. Fraud prevention programs that increase social engagement and reduce behaviors associated with fraud exposure are promising targets for intervention. Results also suggest that embedding fraud awareness education in locations where lottery tickets are sold may be a prevention strategy to reach vulnerable older adults.

